# The Effect of Dietary Protein Imbalance during Pregnancy on the Growth, Metabolism and Circulatory Metabolome of Neonatal and Weaned Juvenile Porcine Offspring

**DOI:** 10.3390/nu13093286

**Published:** 2021-09-20

**Authors:** Miriama Sciascia, Cornelia Prehn, Jerzy Adamski, Gürbüz Daş, Iris S. Lang, Winfried Otten, Solvig Görs, Cornelia C. Metges

**Affiliations:** 1 Institute of Nutritional Physiology ‘Oskar Kellner’, Research Institute for Farm Animal Biology (FBN), 18196 Dummerstorf, Germany; sciascia@fbn-dummerstorf.de (M.S.); gdas@fbn-dummerstorf.de (G.D.); kandera@fbn-dummerstorf.de (I.S.L.); goers@fbn-dummerstorf.de (S.G.); 2 Metabolomics and Proteomics Core (MPC), Helmholtz Zentrum München, German Research Center for Environmental Health, 85764 Neuherberg, Germany; prehn@helmholtz-muenchen.de; 3 Institute of Experimental Genetics, Helmholtz Zentrum München, German Research Center for Environmental Health, 85764 Neuherberg, Germany; adamski@helmholtz-muenchen.de; 4 Department of Biochemistry, Yong Loo Lin School of Medicine, National University of Singapore, Singapore 117597, Singapore; 5 Institute of Biochemistry, Faculty of Medicine, University of Ljubljana, 1000 Ljubljana, Slovenia; 6 Institute of Behavioural Physiology, Research Institute for Farm Animal Biology (FBN), 18196 Dummerstorf, Germany; otten@fbn-dummerstorf.de; 7 Chair of Nutritional Physiology and Animal Nutrition, Faculty of Agriculture and Environmental Sciences, University of Rostock, 18059 Rostock, Germany

**Keywords:** maternal protein restriction, offspring, body weight, carbohydrate and urea metabolism, metabolomics, porcine model

## Abstract

Protein imbalance during pregnancy affects women in underdeveloped and developing countries and is associated with compromised offspring growth and an increased risk of metabolic diseases in later life. We studied in a porcine model the glucose and urea metabolism, and circulatory hormone and metabolite profile of offspring exposed during gestation, to maternal isoenergetic low–high (LP-HC), high–low (HP-LC) or adequate (AP) protein–carbohydrate ratio diets. At birth, LP-HC were lighter and the plasma acetylcarnitine to free carnitine ratios at 1 day of life was lower compared to AP offspring. Plasma urea concentrations were lower in 1 day old LP-HC offspring than HP-LC. In the juvenile period, increased insulin concentrations were observed in LP-HC and HP-LC offspring compared to AP, as was body weight from HP-LC compared to LP-HC. Plasma triglyceride concentrations were lower in 80 than 1 day old HP-LC offspring, and glucagon concentrations lower in 80 than 1 day old AP and HP-LC offspring. Plasma urea and the ratio of glucagon to insulin were lower in all 80 than 1 day old offspring. Aminoacyl-tRNA, arginine and phenylalanine, tyrosine and tryptophan metabolism, histidine and beta-alanine metabolism differed between 1 and 80 day old AP and HP-LC offspring. Maternal protein imbalance throughout pregnancy did not result in significant consequences in offspring metabolism compared to AP, indicating enormous plasticity by the placenta and developing offspring.

## 1. Background

The Developmental Origins of Health and Disease hypothesis proposes that exposure of the developing fetus to a hostile uterine environment during critical phases of growth and development can significantly impact the offspring’s short- and long-term health [1,2]. Inadequate nutrition during pregnancy, both in composition and amount, is a significant stressor on the developing embryo/fetus as the mother is the sole source of nutrients. In underdeveloped and developing countries, access to sufficient amounts/quality protein sources is difficult and has become a major public health problem [3,4]. Studies have shown that exposure to maternal protein undernutrition during fetal development can affect the structural and functional features of offspring and lead to an increased propensity for chronic disease [2]. In addition, human and animal studies have shown that it is not just maternal protein undernutrition that negatively impacts fetal development, but protein excess too [5,6,7,8,9].

The majority of animal studies examining the effect maternal protein imbalance on offspring development, have been performed in rodents, which, at birth, are developmentally less mature than human infants [10]. In many of these rodent studies the effect of maternal diet composition on offspring development and health, semi-synthetic diets were used [11,12,13]. However, it remains controversial whether these models adequately reflect the transgenerational effects of diet. Additionally, exposure has been conducted during both gestation and lactation [13,14,15,16], complicating analysis of how in utero exposure is linked to the onset of metabolic diseases later in life.

The pig is a well-established animal model for studying the effect of maternal nutrition on offspring metabolism in humans [17,18,19], whose post-natal development is closely related to that of humans [20,21]. Thus, we developed a model of intrauterine growth restriction using a modest dietary protein imbalance, in first-pregnancy sows, by feeding them isoenergetic [22] low protein to high carbohydrate (LP-HC; 1:10.4), high protein to low carbohydrate (HP-LC; 1:1.3) or adequate (AP; 1:5) diets, throughout pregnancy. Offspring from all three diet groups were then cross-fostered to sows fed an AP diet throughout, to remove any influence the pregnancy diet may have on post-natal development. Using this model, we have reported that the modulated protein to carbohydrate ratios result in altered fetal cortisol regulation (LP-HC) [6], birth weight, body composition, mitochondrial biogenesis and gene methylation (LP-HC), adipocyte energy processes and lipid transport, hepatic cell cycle and proliferation (LP-HC, HP-LC), and muscle filament and cell cycle progression in the offspring (HP-LC) [22,23,24,25,26,27,28,29]. Several of these companion studies assessed the impact of offspring age on the hepatic and muscle transcriptome and found that age had a stronger effect than maternal diet, implying considerable offspring resilience towards exposure to a mildly imbalanced maternal protein diet in utero [26,27,28,29].

Metabolomics is a tool that can provide a snapshot in time of biological processes underpinning experimentally observed phenotypic, proteomic and transcriptomic changes [30]. The circulatory metabolite and hormone profiles can be most informative as they reflect systemic changes (interactions from all the organs affected) by, for example, the nutritional insult used in a dietary model of protein imbalance. Metabolomics has been applied to a variety of studies in pigs [31,32,33], but only few exploring the plasma metabolome of neonatal piglets [34,35]. Therefore, the objectives of this study were twofold: (1) First, we were interested to know whether exposure to a moderate dietary protein imbalance in utero, known to moderately reduce neonatal birth weight, would also affect the metabolism of offspring during the juvenile stage. (2) Second, whether the investigation of plasma metabolite profiles and metabolic function in porcine offspring exposed to maternal protein imbalance during pregnancy could shed light on the previously reported proteomic, transcriptomic and phenotypic changes in this model [22,24,25,26,27,34,35]. Therefore, we characterized the glucose and urea metabolism, and circulatory hormone and metabolite profile of porcine offspring exposed to a maternal LP-HC and HP-LC diets during gestation, and explored the associations with the different phenotypic, proteomic and transcriptomic data previously reported with this porcine model of mild dietary protein imbalance.

## 2. Methods

### 2.1. Experimental Pregnancy Diets

Experimental pregnancy diets were formulated to have an adequate (AP; 12.1% crude protein, 3.7% crude fat, 7.4% crude fiber), low (LP-HC; 6.5% crude protein, 2.9% crude fat, 8.9% crude fiber) or high (HP-LC; 30% crude protein, 3.7% crude fat, 10.3% crude fiber) protein concentration [22]. Diets were isoenergetic (13.7 MJ of metabolizable energy/kg) with a protein to carbohydrate ratio of 1:5, 1:10.4 and 1:1.3, respectively. This required the addition of crystalline L-amino acids (AA) to the LP-HC and HP-LC diets to achieve AA proportions similar to the AP diet. In order to test and compare practical diets under real-world conditions we refrained from semi-synthetic diets and extremely low or high protein levels as previously reported [36,37,38]. However, it should be noted that the low and high protein levels used in the present study are clearly below and in excess of the required dietary protein level, respectively, as recommended for primiparous sows [39]. In the LP-HC group, the intake of indispensable AA was 50% less than recommendations, whereas in the HP-LC group, gilts consumed ~250% of indispensable AA, as compared with the AP group [22]. As the experimental diets were isoenergetic, the different dietary protein levels were balanced by different levels of carbohydrates and fat. Thus, the content of digestible and non-digestible carbohydrates and fat in LP-HC/HP-LC differed from the (AP) control diet.

**Figure 1:**
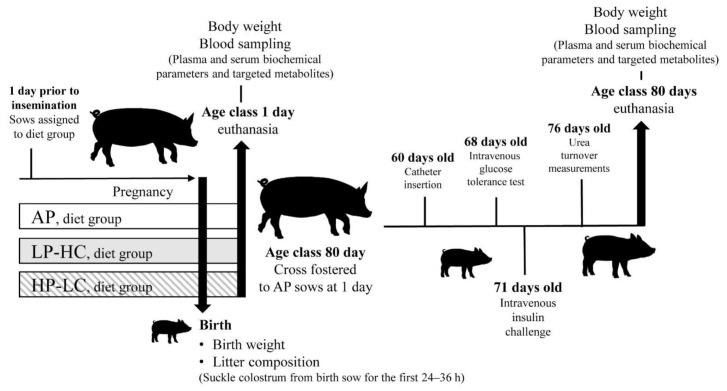
Experimental design. Primiparous sows were fed low (LP-HC; 1:10.4, *n* = 18), high (HP-LC; 1:1.3, *n* = 16) or adequate (AP; 1:5, *n* = 17) protein to carbohydrate ratio diets from 1 day prior to insemination until birth.

Gilts were fed the experimental diets twice daily (07:00 and 15:00 h) throughout pregnancy (Figure 1) and had free access to water.

### 2.2. Animals and Postnatal Offspring Diets

The animal study was conducted from 2005 to 2008, with all animal care and experimental procedures conducted in accordance with the German Animal Welfare Act approved by the State Office for Agriculture, Food Safety and Fishing Mecklenburg-Western Pomerania, Germany (7221.3–1.1–006/04; 7221.3–1.2–05/06; 7221.3–1.2–013/06).

German Landrace sows were sourced from the experimental pig facility of the Research Institute for Farm Animal Biology. One day prior to insemination, gilts (*n* = 93) were randomly assigned to one of three pregnancy diets: AP, LP-HC or HP-LC. At 115 days post insemination labor was induced, as previously described [22]. Litter characteristics were recorded immediately after birth and detailed results reporting gilt development, feed intake and colostrum composition of the entire cohort have been previously published [22].

At farrowing, experimental male and female (AP = 80, LP-HC = 74, HP-LC = 85) offspring from 51 litters (AP = 17, LP-HC = 18, HP-LC = 16) spread across 8 experimental blocks were selected for the current study (Figure 1). For the first 24–36 h of life, all experimental piglets remained with their mother to ensure sufficient uptake of colostrum. Within 36 h after birth, age class 1 day piglets (AP, *n* = 53; LP-HC, *n* = 49; HP-LC, *n* = 58) were euthanized with T61 (Tetracaine hydrochloride 5 mg/mL, mebezonium iodide 50 mg/mL, Embutramid 200 mg/mL, Intervet Deutschland GmbH, Unterschleißheim, Germany). While age class 80 day piglets (AP = 27, LP-HC = 25, HP-LC = 27) were cross-fostered to standardized litters (11 piglets/sow) from sows (parity 2–4) fed AP pregnancy and standard lactation diets (Figure 1). This was done to remove potential postnatal effects of gilt experimental diet and litter size on measurements taken after the colostrum intake period (24–36 h post birth). At day 6 of life, all experimental male piglets were castrated. Post-weaning (day 28 of life) piglets had ad libitum access to the same standard commercial diets [40]. From day 28 of life, piglets were provided with a starter diet (Turbostart, 15 MJ ME/kg; Trede & von Pein, Dammfleth, Germany) for 4 days, before being introduced to a conventional pig starter diet (Porcistart 14 MJ ME/kg; Trede & von Pein, Dammfleth, Germany). At day 48, piglets were switched to a grower diet (Porcibig; 13.8 MJ ME/kg; Trede & von Pein, Dammfleth, Germany) until day 76 when they were provided with Vormast Cafo Top (13.6 MJ ME/kg; Trede & von Pein, Dammfleth, Germany). At day 79, offspring were fasted overnight for 12 h and then euthanized via exsanguination after electro-stunning at day 80 (Figure 1).

Offspring body weight (BW) for both age classes was determined at birth and at euthanasia. For age class 80 day, body weight was also recorded at days 28, 35, 42, 49 and 56, and on day 68 (intravenous glucose tolerance test (IVGTT) and glucose turnover test), day 71 (intravenous insulin challenge (IIC) and day 76 (urea turnover).

### 2.3. Blood Sampling

At euthanasia, blood samples were collected into Li–Heparin tubes (Sarstedt, Germany) for both age classes and centrifuged for 20 min at 1576× *g* and 4 °C to obtain plasma. A subset (age class 1 day: AP, *n* = 23; LP-HC, *n* = 28; HP-LC, *n* = 18; age class 80 day: AP, *n* = 13; LP-HC, *n* = 14; HP-LC, *n* = 7) of blood samples from blocks 1–4 was collected into Serum Z tubes (Sarstedt, Germany), and left at room temperature for 2 h to coagulate and obtain serum. Plasma and serum samples were stored at −80 °C for subsequent clinical chemistry and targeted metabolite analysis. Clinical chemistry samples were analyzed within three months after collection, while targeted metabolite samples were analyzed eight years after collection. We have previously shown that the overall stability of the metabolites stored at −80 °C for five years is very good [41], and our own data (unpublished) shows that 7 years has the same low or non-impact. At day 60, the age class 80 d offspring were surgically fitted with an indwelling jugular catheter, to allow frequent blood sampling as previously described [42]. All offspring were fasted for 16 h overnight and a basal blood sample was collected 15 and 5 min prior to the start of each experimental protocol. At day 68, an IVGTT and glucose turnover measurements were conducted. Blood samples were collected 2, 3, 5, 8, 10, 15, 20, 30, 40, 60 and 240 min post injection and treated according to the work in [42]. The resulting plasma samples were stored at −20 °C, and subsequent analyses performed within 1 year of collection. At day 71, an IIC was performed, and blood samples were taken 5, 10, 15, 30, 45, 60, 75, 90, 120, 150, and 180 min relative to dosing. At day 76, the same offspring were utilized for urea turnover measurements and blood samples were collected 5, 10, 15, 20, 30, 60, 90, 120, 150, 240 and 360 min post injection. Blood samples were treated according to [42] and the resulting plasma samples were stored at −80 °C for subsequent analyses. Intravenous insulin challenge samples were analyzed within 3 months of collection, while urea turnover samples were analyzed within one year of sample collection.

### 2.4. Plasma and Serum Biochemical Parameters

Plasma glucose, non-esterified fatty acids (NEFA), triglycerides, total cholesterol and urea concentrations were measured at the University of Veterinary Medicine Hannover, Germany [42]. Serum high-density lipoprotein (HDL) and low-density lipoprotein (LDL) cholesterol concentrations were measured using HDL (650207) and LDL (969706) cholesterol kits from Beckman Coulter GmbH (Krefeld), at the Institute for Clinical Chemistry and Laboratory Medicine, University of Rostock. Plasma insulin (PI–12K) and glucagon (GL–32K) were measured by radioimmunoassay using commercially available porcine kits (Biotrend Chemikalien GmbH, Köln, Germany). The minimum detectable concentration as reported by the kit manufacturer was 1.611 μU/mL and 18.453 pg/mL (±2 SD), respectively, while the intra- and inter-assay coefficients of variance were for insulin (8.4% and 9%) and glucagon (4% and 8.1%). The glucagon to insulin ratio was calculated as the glucagon concentration in nmol/L divided by the insulin concentration in nmol/L. The glucose to insulin ratio was calculated as the glucose concentration in mmol/L divided by the insulin concentration in pmol/L.

### 2.5. Intravenous Glucose Tolerance Test/Insulin Challenge and Glucose Turnover Measurements

At day 68, offspring were given a mixed bolus of 0.2 g glucose (Glucose 50, Braun, Melsungen AG, Melsungen, Germany) and 1.5 mg of ^13^C_6_-glucose (99 atom% ^13^C; Berlin–Chemie AG, Berlin, Germany) per kg BW through the jugular catheter, for IVGTT and glucose turnover measurements. Plasma samples were used to determine glucagon, glucose and insulin concentrations for the IVGTT and plasma glucose turnover was calculated according to [43]. Glucose concentrations were measured using a microplate absorbance reader (Sunrise^TM^, Tecan Austria GmbH, Grödig/Salzburg, Austria), while insulin and glucagon concentrations were measured as described in the previous section. The enrichment of ^13^C_6_-glucose was measured by GCMS in positive chemical ionization mode after conversion to aldonitrile pentaacetate derivatives using *m*/*z* 328 (m + 0) and *m*/*z* 334 (m + 6) as diagnostic ions, and the glucose turnover was calculated according to [44]. At day 71, an intravenous insulin challenge was conducted by administering a 0.5 I.U./kg BW bolus of porcine insulin (27 I.U./mg; Sigma-Aldrich, Taufkirchen, Germany), as reported in a companion study [45]. Glucose was determined as described above.

### 2.6. Urea Turnover Measurements

At day 76, pigs were given a bolus injection of 3.5 mg ^15^N_2_–urea (99 At.-% ^15^N, Chemotrade, Leipzig) per kg BW through the jugular catheter. Plasma ^15^N urea enrichment was measured as *tert*–butyldimethylsilyl derivatives by GCMS with positive chemical ionization in selected ion monitoring mode for *m*/*z* 231 to 233 quantification and urea turnover rate was determined [46].

### 2.7. Targeted Metabolomics

Targeted metabolite analysis was conducted using a subset of plasma samples (*n* = 8/diet/age class) which were derived from 36 different litters and balanced for offspring sex. Analysis was based on LC-ESI-MS/MS and FIA-ESI-MS/MS measurements using the AbsoluteIDQ^TM^ p180 Assay Kit (Biocrates Life Sciences AG, Innsbruck, Austria). The assay utilized 10 µL plasma and enabled 188 metabolites to be quantified; 39 acylcarnitines + free carnitine, 21 amino acids (AA; 19 proteinogenic + citrulline + ornithine), 21 biogenic amines, 90 glycerophospholipids (14 lysophosphatidylcholines (lysoPC) and 76 phosphatidylcholines (PC)), 15 sphingolipids and hexoses (sum of hexoses—about 90–95% glucose) simultaneously. The abbreviation Cx:y is used to describe the total number of carbons (x) and double bonds (y) of all chains, respectively [47]. For LC-ESI-MS/MS and FIA-ESI-MS/MS, compound identification and quantification were based on scheduled multiple reaction monitoring measurements. The method of AbsoluteIDQ^TM^ p180 Kit conforms with the EMEA-Guidelines “Guideline on bioanalytical method validation” (21 July 2011) [48], which implies proof of reproducibility within a given error range. Measurements were performed as described by the manufacturer (manual UM-P180). The limit of detection (LOD) was set to three times the value of the zero samples (PBS). The AbsoluteIDQ^TM^ p180 Kit assay procedures, metabolite nomenclature, sample handling and mass spectrometric analyses have been all previously described in detail [47].

### 2.8. Data Evaluation and Statistical Analysis

Offspring body weight data were analyzed using a mixed model that contained the fixed effects of maternal diet, experimental block, offspring sex, litter size class (litters <13 and ≥13 piglets according to 13 as the median of litter size), the random factor gilt nested in maternal diet × experimental block (1–8) × litter size class and the interactions of maternal diet × offspring sex and maternal diet × litter size class. Clinical blood parameters, glucose and urea turnover data and fasted analyte values prior to IVGTT were analyzed with a mixed model using the fixed effects of maternal diet, offspring age class and sex, with the additional fixed effects of experimental block and litter size class, and the random factor gilt nested in maternal diet × experimental block × litter size class × offspring age class, and the interactions of maternal diet × offspring sex × offspring age class, maternal diet × offspring age class and offspring sex × offspring age class. The model for IVGTT and IIC were analyzed with a mixed model containing the fixed effects of maternal diet, offspring sex, litter size class, the random factor gilt nested in maternal diet × experimental block × litter size class × time, the interactions of maternal diet × offspring sex, maternal diet × litter size class, maternal diet × time, maternal diet × offspring sex × time and maternal diet × litter size class × time and an exponential spatial model (SP(EXP)) to account for unequally spaced repeated measurements. Metabolite ratio and pool data were analyzed using a model that contained the fixed effects of maternal diet, offspring age class, sex and the interactions of maternal diet × offspring sex × offspring age class, maternal diet × offspring age class and offspring sex × offspring age class. All statistical analyses were conducted in SAS 9.4 (Copyright, SAS Institute Inc., Cary, NC, USA) and for all models, least squares means (LSmeans) and their standard error (SE) were computed for each fixed effect and analyzed with Tukey’s multiple comparison post hoc tests. Concentrations are reported as LSmean values ± SE. Significance is reported as *p* ≤ 0.05.

Targeted metabolite data evaluation for quantification and quality assessment was performed with the software packages MultiQuant 3.0.1 (Sciex Deutschland GmbH, Darmstadt, Germany) and MetIDQ™ (Biocrates Life Sciences AG). Internal standards served as references for the calculation of metabolite concentrations (µM). Out of the 188 metabolites, 42 were excluded from all diet (AP, HP-LC and LP-HC) and age classes (1, 80 d) and subsequent analyses due to concentrations being below the LOD or lower limit of quantification (Supplementary Table S1). Metabolite data were then analyzed with the use of MetaboAnalyst 5.0 [49]. Data were generalized logarithm (glog) transformed and quantile normalized prior to statistical analysis. Datasets were first analyzed by principal component analysis to lower data dimensionality and to acquire an overview by presenting trends, groupings, and classify potential outliers. Samples were considered as outliers when they were situated outside the 95% confidence ellipse region of the model. The separation between each diet group (AP v. HP-LC v. LP-HC) and age class (1 v. 80 d) was visualized using partial least-squares discriminant analysis (PLS-DA), which is a common tool to perform classification and regression in metabolomics. The method uses multivariate regression techniques to optimize separation between different groups. The quality of the PLS-DA model was verified by leave-one-out cross-validation using two performance indicators: Q^2^ (0.40 > Q^2^ < 1.0), “goodness of prediction”, or predicted variation and R^2^ (0.75 > R^2^ < 1.0), known as the goodness of fit, or explained variation. In addition, classification accuracy is also reported. Statistical significance of metabolite differences was assessed with paired parametric t-test with false discovery rates (FDR; q-value) correction. The FDR *p* values less than 0.05 were considered statistically significant. Metabolic pathway enrichment analysis was performed using MetaboAnalyst 5.0. The human metabolome database (HMDB) ID of significantly different compounds was uploaded and embedded in the human pathway library for pathway analysis and hypergeometric tests, with pathway analysis algorithms of the Fisher’s exact test, topology algorithms of relative betweenness centrality, and Kyoto encyclopedia of genes and genomes (KEGG) pathway library version of Homo sapiens. Calculation of relevant metabolite ratios and pools was performed using the MetIDQ^TM^ RatioExplorer software package (Biocrates Life Sciences AG). This package is a module that provides a set of preconfigured sums and ratios that have been reported to be informative in clinical or pathophysiological studies (Supplementary Table S2).

## 3. Results

### 3.1. Litter Characteristics and Offspring Body Weight

Characteristics for each litter used in the current study (experimental + non-experimental piglets; AP, *n* = 197; LP-HC, *n* = 167; HP-LC, *n* = 196) were assessed. Results showed that the average piglet body weight, female and male offspring body weight and average live born piglet body weight were lower from LP-HC compared to AP gilts (Supplementary Table S3). No differences were observed in the other litter characteristics assessed (Supplementary Table S3).

In the population of experimental piglets used in this study (AP, *n* = 53; LP-HC, *n* = 49; HP-LC, *n* = 58), LP-HC offspring were significantly lighter than AP (*p* = 0.04) at day 1 of life, and HP-LC (*p* = 0.02) were significantly lighter than AP offspring at 80 days of life (Table 1). There was a significant effect of litter size (*p* = 0.01) and the interaction of maternal diet × litter size (*p* = 0.05) on body weight at day 1 of life. While maternal diet (*p* = 0.03) had a significant effect on offspring body weight at 80 day of life. No other body weight differences were observed (Supplementary Table S4).

### 3.2. Plasma and Serum Biochemical Parameters

At day 1 of life, the concentration of urea was significantly (*p* = 0.04) lower in LP-HC than HP-LC offspring (Table 1). Comparisons between the two offspring age classes within each maternal diet group showed the concentration of plasma triglycerides was lower (*p* = 0.05) in neonatal compared to 80 day old HP-LC offspring. The concentration of urea (AP, LP-HC, HP-LC; *p* < 0.001), glucagon (AP, HP-LC; *p* < 0.001) and the ratio of glucagon to insulin (AP, LP-HC; *p* < 0.001, HP-LC; *p* < 0.001) was lower in neonatal compared to 80 day old offspring, in all three maternal diet groups (Table 1). There was a significant effect of offspring age class on total cholesterol (*p* < 0.05; (Supplementary Table S5), triglycerides, urea, glucagon and the ratio of glucagon to insulin (*p* < 0.001; Table 1). No other differences were observed (Supplementary Table S5).

### 3.3. Intravenous Glucose Tolerance, Insulin Challenge and Turnover Studies

**Figure 2:**
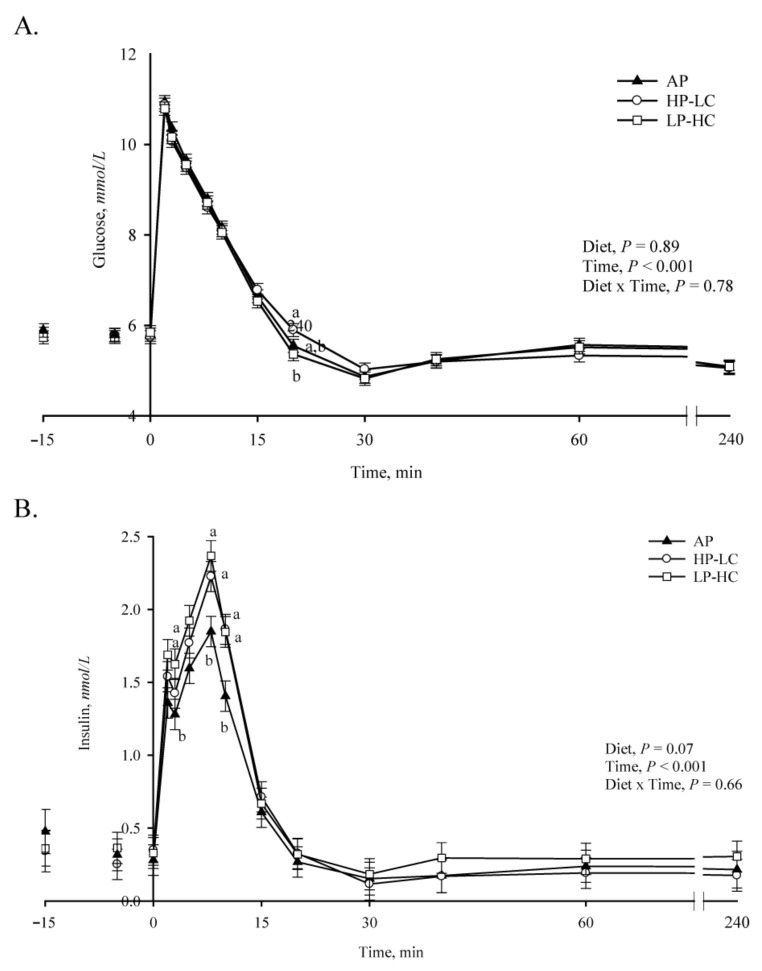
Intravenous glucose tolerance test conducted at 68 days of life in juvenile porcine offspring exposed to low (LP-HC; 1:10.4, *n* = 25), high (HP-LC; 1:1.3, *n* = 27), or adequate (AP; 1:5, *n* = 25) protein to carbohydrate ratio diets during gestation. Plasma concentrations of (**A**) glucose and (**B**) insulin. Values are LSmeans ± SE; means without a common letter differ (*p* < 0.05), based on Tukey post hoc analysis.

At 68 days of age, offspring were weighed and an IVGTT conducted. Plasma glucagon, glucose and insulin concentrations were monitored, and ratios calculated. Glucose injection induced hyperglycemia, which returned to basal values between 15–20 min, and at 20 min LP-HC offspring had lower plasma glucose concentrations compared to HP-LC (*p* = 0.03, Figure 2A). At 3, 8 and 10 min, insulin concentrations were significantly (*p* ≤ 0.05) higher in both LP-HC and HP-LC compared to AP offspring (Figure 2B). No difference in plasma glucagon (Supplementary Figure S1A), the glucose to insulin ratio (Supplementary Figure S1B) or plasma glucagon to insulin ratio (Supplementary Figure S1C), or fasting glucagon, glucose and insulin concentration values (Supplementary Table S6) was observed. Additionally, no difference in body weight, the glucose or insulin area under the curve, glucose turnover or pool size was observed (Supplementary Table S6). No difference in body weight (data not shown), fasting glucose levels or response to the insulin challenge were observed in offspring at day 71 (Supplementary Figure S2). No difference in urea turnover was observed at day 76 (Supplementary Table S7).

### 3.4. Targeted Metabolite Profiles

**Figure 3:**
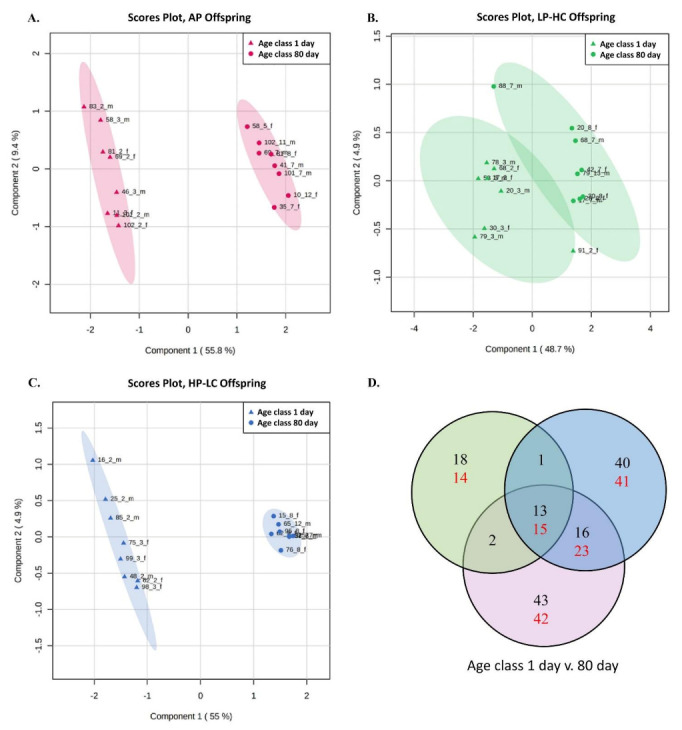
Partial least square discriminate analysis (PLS-DA) and significantly different metabolites between age class 1 and 80 day porcine offspring exposed to low (LP-HC; 1:10.4), high (HP-LC; 1:1.3) or adequate (AP; 1:5) protein to carbohydrate ratio diets during gestation. PLS-DA plot for (**A**) AP (Measure, 3 comps; Accuracy, 1; R^2^ = 0.97; Q^2^ = 0.98), (**B**) LP-HC (Measure, 1 comp; Accuracy, 0.875; R^2^ = 0.59; Q^2^ = 0.41), (**C**) HP-LC (Measure, 5 comps; Accuracy, 1; R^2^ = 1; Q^2^ = 0.97) and (**D**) the number of differently abundant metabolites between the different offspring (black, higher; red, lower, in age class 80 compared to 1 day offspring). Red, green and blue symbols and areas denote the data of offspring exposed to AP, LP-HC and HP-LC offspring, respectively.

Partial Least Squares-Discrimination Analysis was utilized to clarify any potential discrimination between the two-way group comparisons. The result of each comparison showed this discrimination could only be made for age class (1 vs. 80 day) comparisons within each diet group; AP, (Measure, 3 comps; Accuracy, 1; R^2^ = 0.97; Q^2^ = 0.98, Figure 3A), LP-HC (Measure, 1 comp; Accuracy, 0.88; R^2^ = 0.59; Q^2^ = 0.41, Figure 3B) and HP-LC (Measure, 5 comps; Accuracy, 1; R^2^ = 1; Q^2^ = 0.97, Figure 3C). 

Analysis of the individual metabolites, and relevant metabolite ratios and sums between the different diet groups within each offspring age class showed that the C2/C0 (acetylcarnitine to free carnitine) ratio was lower in 1 day old LP-HC compared to AP offspring (Supplementary Table S8).

A comparison between the age classes within each diet group, identified for AP; 85 (43 increased/42 decreased), HP-LC; 81 (40 increased/41 decreased) and LP-HC offspring; 32 (18 increased/14 decreased) significantly different individual metabolites (Figure 3D) in 80 compared to 1 day. Subsequent analyses showed that 28 (13 increased/15 decreased) significantly different metabolites were shared between all three diet groups, 39 (16 increased/23 decreased) between AP and HP-LC groups, 2 (both increased) between AP and LP-HC and 1 (increased) between LP-HC and HP-LC groups (Figure 3D) in 80 compared to 1 day. Furthermore, 16 metabolites unique to the AP group were identified, whilst HP-LC had 13 and LP-HC had 1.

Pathway analysis via KEGG identified significant changes in aminoacyl-tRNA, arginine and phenylalanine, tyrosine and tryptophan biosynthesis, histidine and beta-alanine metabolism in AP and HP-LC offspring, between age classes 1 and 80 day (Table 2). An analysis of the significantly different metabolites shared between the AP and HP-LC offspring showed significant changes in aminoacyl-tRNA, phenylalanine, tyrosine and tryptophan biosynthesis, histidine and beta-alanine metabolism, between 1 and 80 d (Table 2).

Analysis of the relevant metabolite ratios and sums between the different age classes within each diet group showed that for AP offspring, the C2/C0, Fischer’s ratio and sum of aromatic AA; for HP-LC offspring, the C2/C0, citrulline/ornithine, Fischer’s ratio and sum of aromatic AA; and for LP-HC offspring, the ratios of C2/C0 and symmetric dimethylarginine/arginine were different (Supplementary Table S8).

## 4. Discussion

The aim of this study was to investigate alterations in the hormone and metabolic processes of newborn and weaned juvenile porcine offspring exposed to protein imbalanced diets during the in utero period only. This is in contrast with previous studies in rodents where imbalanced diets were fed during pregnancy and lactation [11,13]. This complicates separation of effects of in utero and early postnatal dietary exposure in the offspring. We have previously shown in mice that a high protein diet fed to the dams solely during lactation has a different effect on pre-weaning offspring growth and the liver transcriptome, than in utero dietary exposure to the same diet [50,51]. Furthermore, as we did not use semi-synthetic diets but real feedstuffs to design isoenergetic diets, the three diets were not completely comparable in the levels of digestible and non-digestible carbohydrates, as well as fat. Thus, we conclude that the effects LP-HC and HP-LC diets had on the dams’ metabolism (and indirectly on the offspring) was a shift in the intermediary macronutrient metabolism as reported earlier [5,42,52]. However, note that the composition of the non-digestible carbohydrates is unknown. Therefore, it is not known to what extent the differences in fiber may have influenced the outcome of this study.

Low-protein diets fed during pregnancy have been shown to reduce offspring birth weight in several species [13,14,36]. Results from the current study show that exposure to a low-protein diet in utero significantly reduced the average birth weight of all live born, female and male piglets by 15%, compared to AP offspring. Fetal growth restriction in the current study was however much less severe than the 33 and 35% birth weight reduction previously reported for maternal low protein diets throughout pregnancy with 0.5% protein [36,38]. In the sub-population of LP-HC and HP-LC offspring selected from the litters for the growth, circulating metabolome and metabolic flexibility sections of this study, there was no significant difference in birth weight. However, results from the complete study population published in a companion paper show moderately lower birth weight in LP-HC as well as in HP-LC litters compared to AP [22]. By 1 day of age, LP-HC were lighter than AP offspring. From birth to 1 day of age, the only nutrient source was colostrum from the birth mother, and body weight gain in the first days of life is associated with colostrum quality [53], yield and intake [54]. Colostrum quality from the gilts in the current study showed no significant difference in lactose, fat and protein concentrations between the three pregnancy diet groups [22]. While colostrum yield was not measured in the current study, it has been reported that maternal protein restriction lowers colostrum yield and colostrum yield was positively correlated with the concentration of plasma urea on the day before farrowing [55]. In a companion paper, we reported that gilt and fetal plasma urea concentrations of HP-LC were significantly higher, and LP-HC significantly lower compared to AP, at 94 days of pregnancy [5]. In addition, results from the clinical blood parameters reported in the current study show that 1 day old LP-HC offspring had lower plasma urea concentrations compared to HP-LC. We have also reported in another companion paper that Galactokinase 1 gene expression in the liver of 1 day old LP-HC offspring was lower compared to AP, reflecting decreased galactose supply due to low milk supply [24]. Thus, taken together these results suggest that the lower body weight of 1 day old LP-HC offspring was potentially linked to reduced colostrum yield/intake, whilst the lower plasma urea concentration was a potential carryover effect from intrauterine environment.

In the current study, metabolic flexibility was assessed via IVGTT, IIC and glucose and urea turnover in juvenile offspring only. Results from the IVGTT showed that insulin levels were significantly higher during the first 10 min post glucose bolus injection in both LP-HC and HP-LC piglets, indicating offspring from protein malnourished gilts required more insulin to metabolize glucose than AP offspring. However, results from the IIC showed no difference between offspring from the three dietary maternal groups. Thus, it appears that glucose turnover in offspring from gilts fed a protein imbalanced diet may be more closely related to greater insulin secretion than to differences in whole-body sensitivity to insulin. Intravenous glucose tolerance tests in rodents exposed to maternal protein restriction during gestation and suckling [15] or late-gestation [16], support this observation and hypothesize it is linked to reduced pancreatic mass [56] and altered pancreatic β cell function, resulting in reduced expression of specific insulin-signaling proteins. We have previously reported reduced pancreatic mass in the juvenile LP-HC and HP-LC offspring used in the current study, suggesting a similar mechanism maybe responsible for insulin regulation of glucose metabolism in these animals [57].

A review of companion studies investigating phenotypic, transcriptome (liver and muscle) and proteome (subcutaneous adipose tissue) changes in the AP, LP-HC and HP-LC offspring used in the current study was conducted [5,22,40,42,57]. It showed that there were a significant number of differences observed in the muscle [29] and liver [28] of HP-LC compared with AP offspring, and subcutaneous adipose tissue [25] of HP-LC and LP-HC compared to AP, at 1 day of life, while significant differences in the liver of LP-HC were also observed compared to AP offspring at 80 days of life [58]. However, although the transcriptome and proteome differences were significant, their biological significance is moderate, as the phenotypic differences were only modest. Yet, in the current study, the targeted metabolomics performed on a sub-population of 1 and 80 day old LP-HC, HP-LC and AP offspring identified only one difference between the offspring from the three gestation diet groups; the C2/C0 (acetylcarnitine to free carnitine) ratio, which was lower in LP-HC compared to AP, at 1 day of life. This almost complete absence of significant differences was unexpected, and indicates three potential hypotheses, that (1) there is a compensatory role of the placenta ameliorating the metabolic effects the maternal diets have on the dams, as observed for fetal plasma amino acid profiles suggesting only a mild in utero insult [5]. (2) There is significant post-natal adaption by the organs of the offspring by adequate nutrient supply via milk to maintain a stable circulating metabolome, and/or (3) the plasma metabolites investigated are not very informative under constant and adequate dietary conditions.

Another interesting result from the companion transcriptomic studies was that offspring age had a greater impact on observed differences than the maternal diet groups within each age class [26,27,28,29], suggesting considerable resilience of offspring towards the exposure to a mildly imbalanced maternal protein diet in utero. Thus, a similar assessment was performed in the current study. Analyses revealed that AP and HP-LC offspring had the largest number of differences between age class 1 and 80 day, which was reflected in their clear PLS-DA separation, while LP-HC had less than half the number of differences, with minimal PLS-DA separation. These results indicate that the plasma metabolome of LP-HC offspring changed the least between the 1 and 80 day classes. This was further highlighted after KEGG pathway analysis could only identify significant pathway changes for HP-LC and AP offspring. Interestingly, the same five amino acid-related KEGG pathways—aminoacyl-tRNA, arginine biosynthesis, phenylalanine, tyrosine and tryptophan biosynthesis, histidine and beta-alanine metabolism—were identified in AP and HP-LC offspring. In addition, the individual metabolites that significantly changed in each of the five pathways changed (increased or decreased) in an identical manner. As phenylalanine and tryptophan are essential amino acids, identified changes in this pathway were linked to tyrosine synthesis from phenylalanine, while histidine metabolism appeared to support the changes in arginine biosynthesis and overlap with changes in beta-alanine metabolism. Thus, taken together, these results suggest that common metabolic pathways were regulated in a similar manner between 1 and 80 day age classes of AP and HP-LC offspring, and may explain why the metabolite sums of aromatic AA and Fischer’s ratio changed in only HP-LC and AP offspring. The significance of these changes between 1 to 80 day age classes of AP and HP-LC but not in LP-HC offspring is unclear as this is the first study to identify common pathways regulated by offspring exposed to HP-LC and AP diets in utero. One potential explanation for the lack of age differences in the LP-HC offspring metabolome could be that the metabolism of LP-HC offspring is less mature than in the other two groups, or—described another way—that the developmental program of HP-LC offspring is comparable to that of control (AP) offspring, but not that of LP-HC offspring.

## 5. Conclusions

In this study, we show that pre-natal exposure to an LP-HC or HP-LC maternal diet is associated with moderate changes in the circulatory metabolite profile related to glucose and protein metabolism at 1 and 80 days of life. Offspring age was shown to have the greatest impact. Similar pathway regulation between 1 and 80 day old HP-LC and AP offspring was identified, indicating that, metabolically, these two groups were more similar than LP-HC, and potentially LP-HC offspring were metabolically less mature.

The main contribution of our study is the finding of considerable metabolic resilience and plasticity in the offspring when “real” maternal diets with only moderate protein deficiency or excess are supplied during gestation. Furthermore, the blood plasma metabolome as a reflection of metabolic adaptations to moderate maternal in utero protein imbalance seems to indicate only a mild in utero insult. Another possible explanation would be that negative consequences of a nutritional insult during prenatal life can be largely resolved if the nutrient supply is adequate during the suckling period. Thus, our observations may provide clues for the design of future meaningful studies examining the mid- and long-term transgenerational effects of maternal pregnancy and/or lactation diets.

## Figures and Tables

**Table 1:** Body weight, plasma and serum biochemical parameters of 1 and 80 day old porcine offspring exposed to low (LP-HC; 1:10.4), high (HP-LC; 1:1.3), or adequate (AP; 1:5) protein to carbohydrate ratio diets during gestation.

		Maternal Diet ^1^			*p* Value, ≤ ^2^
Parameters	Age Class ^3^	AP	LP-HC	HP-LC	Age Class
Body weight, kg					
Day 1	1 and 80 day	1.44 ± 0.05 ^a^	1.25 ± 0.05 ^b^	1.33 ± 0.05 ^a,b^	-
Day 80	80 day	31.9 ± 0.87 ^a^	30.6 ± 0.78 ^a,b^	28.1 ± 1.10 ^b^	
Metabolites, mmol/L					
Triglycerides	1 day	0.78 ± 0.11	0.79 ± 0.11	0.95 ± 0.11 ^c^	<0.001
	80 day	0.42 ± 0.12	0.41 ± 0.12	0.50 ± 0.12 ^d^	
Urea	1 day	5.05 ± 0.37 ^a,b,c^	4.01 ± 0.36 ^a,c^	5.61 ± 0.35 ^b,c^	<0.001
	80 day	2.22 ± 0.40 ^d^	2.20 ± 0.42 ^d^	2.17 ± 0.41 ^d^	
Hormones					
Glucagon, pg/mL	1 day	243 ± 35 ^c^	198 ± 35	296 ± 34 ^c^	<0.001
	80 day	68 ± 41 ^d^	50 ± 40	52 ± 42 ^d^	
Insulin, µU/mL	1 day	6.97 ± 1.60	3.31 ± 1.57	9.02 ± 1.52	0.86
	80 day	6.41 ± 1.82	6.92 ± 1.87	6.69 ± 1.85	
Glucagon:Insulin ^4^	1 day	3.17 ± 0.41 ^c^	3.63 ± 0.40 ^c^	2.42 ± 0.39 ^c^	<0.001
	80 day	0.73 ± 0.53 ^d^	0.24 ± 0.50 ^d^	0.50 ± 0.53 ^d^	

^1^ Values are LSmeans ± SE; Labeled LSmeans (^a,b^ *p* ≤ 0.05) without a common letter differ within a row, (^c,d^ *p* ≤ 0.05) without a common letter differ within a column, based on Tukey post hoc analysis. ^2^ ANOVA F-Test. Litter size (*p* = 0.01) and the interaction of Maternal diet × Litter size (*p* = 0.05) had a significant effect on the body weight of 1 day old offspring, while Maternal diet (*p* = 0.03) had a significant effect on the body weight of 80 day old offspring. ^3^ Number of animals; Age class 1 day (AP = 46–53; LP-HC = 47–49; HP-LC = 51–58), except for HDL and LDL cholesterol (AP = 21–23; LP-HC = 28; HP-LC = 15–18). Age class 80 day (AP = 24–27; LP-HC = 23–25; HP-LC = 22–26), except for HDL and LDL cholesterol (AP = 14; LP-HC = 14; HP-LC = 7). ^4^ nmol/nmol.

**Table 2:** KEGG enrichment analyses of differentially abundant metabolites between age class 1 and 80 day porcine offspring exposed to low (LP-HC; 1:10.4), high (HP-LC; 1:1.3) or adequate (AP; 1:5) protein to carbohydrate ratio diets during gestation.

Comparison	KEGG Pathway	Metabolites			
		Identified	Total ^1^	*p* Value	FDR
HP-LC	Aminoacyl-tRNA biosynthesis	10	48	<0.001	<0.001
	Arginine biosynthesis	4	14	<0.001	<0.001
	Histidine metabolism	4	16	<0.001	<0.001
	beta-Alanine metabolism	4	21	<0.001	0.01
	Phenylalanine, tyrosine and tryptophan biosynthesis	2	4	<0.001	0.02
AP	Aminoacyl-tRNA biosynthesis	8	48	<0.001	<0.001
	Arginine biosynthesis	3	14	<0.001	0.03
	Phenylalanine, tyrosine and tryptophan biosynthesis	2	4	<0.001	0.03
	Histidine metabolism	3	16	<0.001	0.03
	beta-Alanine metabolism	3	21	<0.001	0.05
Shared by both	Aminoacyl-tRNA biosynthesis	6	48	<0.001	<0.001
AP and HP-LC	Histidine metabolism	3	16	<0.001	0.02
	Phenylalanine, tyrosine and tryptophan biosynthesis	2	4	<0.001	0.02
	beta-Alanine metabolism	3	21	<0.001	0.03

^1^ Total in pathway.

## Data Availability

All data generated or analyzed during this study are available from the corresponding author on reasonable request.

## Supplementary Materials

The following are available online at https://www.mdpi.com/article/10.3390/nu13093286/s1, Table S1: Excluded metabolites from targeted analysis, Table S2: Predefined ratios or sums of metabolites that can be selected for their potential metabolic significance, Table S3: Litter characteristics at birth, of sows fed low (LP-HC; 1:10.4), high (HP-LC; 1:1.3), or adequate (AP; 1:5) protein to carbohydrate ratio diets during pregnancy, Table S4: Statistically non-significant (*p* > 0.05) body weight measurements, from weaning (28 d) to 56 d (prior to catheter implantation) measured in juvenile age class 80 d porcine offspring exposed to low (LP-HC; 1:10.4, *n* = 25), high (HP-LC; 1:1.3, *n* = 27), or adequate (AP; 1:5, *n* = 25) protein to carbo-hydrate ratio diets during gestation. Values are LSmeans ± SE, Table S5: Birth weight, plasma and serum biochemical parameters of 1 and 80 d old porcine offspring exposed to low (LP-HC; 1:10.4), high (HP-LC; 1:1.3), or adequate (AP; 1:5) protein to carbohydrate ratio diets during gestation. Values are LSmeans ± SE, Table S6: Body weight, IVGTT analytes, glucose turnover and pools of 68 d old porcine offspring exposed to low (LP-HC; 1:10.4), high (HP-LC; 1:1.3), or adequate (AP; 1:5) protein to carbohydrate ratio diets during gestation. Values are LSmeans ± SE, Table S7: Body weight, urea turnover and pool of 76 d old porcine offspring exposed to low (LP-HC; 1:10.4), high (HP-LC; 1:1.3), or adequate (AP; 1:5) protein to carbohydrate ratio diets during gestation. Values are LSmeans ± SE, Table S8: Plasma targeted metabolite ratios and sums of porcine offspring exposed to low (LP-HC; 1:10.4), high (HP-LC; 1:1.3), or adequate (AP; 1:5) protein to carbohydrate ratio diets during gestation. Figure S1: Intravenous glucose tolerance test conducted at 68 days of life in juvenile porcine offspring exposed to low (LP-HC; 1:10.4, *n* = 25), high (HP-LC; 1:1.3, *n* = 27), or adequate (AP; 1:5, *n* = 25) protein to carbohydrate ratio diets during gestation. Plasma concentrations of, A glucagon, and ratios of B glucose to insulin and C glucagon to insulin. Values are LSmeans ± SE. Diet, Time and the interaction of Diet × Time are ANOVA F-Test results, Figure S2: Intravenous insulin challenge conducted in 71 day of juvenile porcine offspring exposed to low (LP-HC; 1:10.4), high (HP-LC; 1:1.3), or adequate (AP; 1:5) protein to carbohydrate ratio diets during gestation. Values are LSmeans ± SE. Diet and the interaction of Diet × Time are ANOVA F-Test results.
