# Supplementary material for: The Effect of Dietary Protein Imbalance during Pregnancy on the Growth, Metabolism and Circulatory Metabolome of Neonatal and Weaned Juvenile Porcine Offspring
Source: Nutrients. 2021 Sep 20;13(9):3286. doi: 10.3390/nu13093286 (PMC8471113; doi:10.3390/nu13093286)

The Effect of Dietary Protein Imbalance during Pregnancy on the Growth, Metabolism and Circulatory Metabolome of Neonatal and Weaned Juvenile Porcine Offspring - Sciascia et al. "Online Supplementary Material"

Supplementary Table S1. Excluded metabolites from targeted analysis

Metabolite class	Metabolite Species	Exclusion[1]	Metabolite class	Metabolite Species	Exclusion[1]
Acylcarnitines	C10	< LLOQ	Biogenic amines	c4-OH-Pro	< LOD
	C10:1	< LOD		DOPA	< LOD
	C10:2	< LOD		Dopamine	< LOD
	C12	< LOD		Histamine	< LOD
	C12-DC	< LOD		Met-SO	< LOD
	C12:1	< LOD		Nitro-Tyr	< LOD
	C14	< LOD		PEA	< LOD
	C16-OH	< LOD		Serotonin	< LLOQ
	C16:1	< LOD		Spermine	< LLOQ
	C16:1-OH	< LOD		Total DMA	< LLOQ
	C16:2	< LOD	Glycerophospholipids	LysoPC a C14:0	< LOD
	C16:2-OH	< LOD		LysoPC a C24:0	< LOD
	C18	< LLOQ		PC aa C26:0	< LOD
	C18:1-OH	< LOD		PC aa C40:1	< LOD
	C5-OH (C3-DC-M)	< LOD		PC ae C30:2	< LOD
	C3	< LOD		PC ae C42:5	< LOD
	C3-OH	< LOD	Sphingolipids	SM C22:3	< LOD
	C3:1	< LOD		SM C26:0	< LOD
	C5	< LOD			
	C5-DC (C6-OH)	< LOD			
	C5-M-DC	< LOD			
	C5:1	< LOD			
	C5:1-DC	< LOD			
	C6 (C4:1-DC)	< LOD			
	C6:1	< LOD			
	C7-DC	< LOD			
	C8	< LOD			
	C9	< LOD			

[1]Limit of detection (< LOD) and lower limit of quantification (< LLOQ).

Supplementary Table S2. Predefined ratios or sums of metabolites that can be selected for their potential metabolic significance.

**Metabolite ratio or sum[1]**	**Identifier**	**Description**
(C2 + C3) / C0	Ratio of short chain acylcarnitines to free carnitine	Measure of overall β-oxidation activity
AAA	Sum of aromatic amino acids	
ADMA / Arg	Fraction of asymmetrically dimethylated Arg of the unmodified Arg pool	Inhibition of NO synthase, associated with endothelial dysfunction and cardiovascular risk in general
BCAA	Sum of branched-chain amino acids	Indicator of short term metabolic control (analogy with insulin muscular resistance)
C2 / C0	Ratio of acetylcarnitine to free carnitine	Measure of β-oxidation of even numbered fatty acids
Cit / Arg	Ratio of Cit to Arg	Activity of nitric oxide synthase
Cit / Orn	Ratio of Cit to Orn	Activity of ornithine carbamoylphosphate transferase
CPT-1 ratio	Ratio of long chain acylcarnitines to free carnitine ([C16+C18]/C0)	Activity of carnitine palmitoyl transferase 1, rate limiting step in the uptake of fatty acids into the mitochondria
Essential AA	Sum of essential amino acids	Indicator of nutritional status
Fischer ratio	Ratio of BCAA to AAA	Indicator of liver damage
Glucogenic AA	Sum of selected glucogenic amino acids (Ala, Gly, Ser)	Indicator of glycolytic vs. gluconeogenic acitivity
Kynurenine / Trp	Ratio of Kynurenine to Trp	Ratio of Trp degradation to kynurenine, indicator of indoleamine 2,3-dioxygenase activity (immunosuppression/tolerance)
Met-SO / Met	Fraction of sulfoxidized Met of unmodified Met pool	Measure of systemic oxidative stress
MUFA (PC)	Monounsaturated glycerophosphocholines	Indicator of nutritional lipid content
MUFA (PC) / SFA (PC)	Ratio of monounsaturated to saturated glycerophosphocholines	Measure of the activity of fatty acid desaturases and indicator of nutritional lipid content
Non-essential AA	Sum of non-essential amino acids	Indicator of metabolic / catabolic state
Orn / Arg	Ratio of Orn to Arg	Activity of arginase
PUFA (PC)	Sum of polyunsaturated glycerophosphocholines	Indicator of nutritional lipid content
PUFA (PC) / SFA (PC)	Ratio of polyunsaturated to saturated glycerophosphocholines	Measure of the activity of fatty acid desaturases and indicator of nutritional lipid content
Putrescine / Orn	Ratio of Putrescine / Orn	Activity of ornithine decarboxylase
SDMA / Arg	Fraction of symmetrically dimethylated Arg of the unmodified Arg pool	Indicator of impaired kidney function/renal failure
Serotonin / Trp	Ratio of Serotonin to Trp	Rate of Trp degradation to Serotonin
SFA (PC)	Sum of saturated glycerophosphocholines	Indicator of nutritional lipid composition
Spermidine / Putrescine	Ratio of Spermidine to Putrescine	Activity of spermidine synthase
Spermine / Spermidine	Ratio of Spermine to Spermidine	Activity of spermine synthase

Total (PC+SM)	Sum of choline containing phospholipids	
Total AA	Sum of all amino acids	
Total AC / C0	Ratio of esterified to free carnitine	
Total AC-DC / Total AC	Fraction of dicarboxyacylcarnitines of the total acylcarnitines	Indicators of ω-oxidation of fatty acids
Total AC-OH / Total AC	Fraction of hydroxylated acylcarnitines of the total acylcarnitines	
Total DMA / Arg	Fraction of dimethylated Arg of the unmodified Arg pool	Activity of protein arginine methyl transferases
Total lysoPC	Sum of lysoglycerophosphocholines	
Total lysoPC / Total PC	Ratio of lysoglycerophosphocholines to glycerophosphocholines	Indicator of phospholipase activity
Total PC	Sum of glycerophosphocholines	
Total PC aa	Sum of diacyl-glycerophosphocholines	
Total PC ae	Sum of glycerophosphocholin plasmalogens	
Total SM	Sum of ceramide phosphocholines (sphingomyelins)	
Total SM / Total (SM+PC)	Fraction of ceramide phosphocholines (sphingomyelins) / of total phospholipid pool	
Total SM / Total PC	Ratio of total ceramide phosphocholines (sphingomyelins) to total glycerophosphocholines	
Total SM-non-OH	Sum of non-hydroxylated ceramide phosphocholines (sphingomyelins)	
Total SM-OH	Sum of hydroxylated ceramide phosphocholines (sphingomyelins)	
Total SM-OH / Total SM-non-OH	Ratio of hydroxylated to non-hydroxylated ceramide phosphocholines (sphingomyelins)	
Tyr / Phe	Ratio of Tyr to Phe	Activity of phenylalanine hydroxylase

[1]From the manufacturer's manual, Biocrates UM-RatioExplorer-1

Supplementary Table S3. Litter characteristics at birth, of sows fed low (LP-HC; 1:10.4), high (HP-LC; 1:1.3), or adequate (AP; 1:5) protein to carbohydrate ratio diets during pregnancy.

	Maternal diet		
Parameter	AP	LP-HC	HP-LC
Number of litters	17	16	18
Number of piglets	197	167	196
Litter size at birth	12.1 ± 0.59	13.0 ± 0.60	11.7 ± 0.57
Stillborn	0.77 ± 0.20	0.75 ± 0.21	0.44 ± 0.19
Live born females, *per litter*	6.94 ± 0.58	6.19 ± 0.59	5.39 ± 0.56
Live born males, *per litter*	4.94 ± 0.66	6.81 ± 0.68	6.11 ± 0.64
Total live born	11.4 ± 0.56	12.3 ± 0.57	11.3 ± 0.54
Body weight, *kg*			
Litter (total)	16.3 ± 0.65	15.0 ± 0.67	14.4 ± 0.63
Piglet (average)	1.37 ± 0.04[a]	1.17 ± 0.04[b]	1.25 ± 0.04[a,b]
Female (average)	1.34 ± 0.04[a]	1.15 ± 0.04[b]	1.21 ± 0.04[a,b]
Males (average)	1.43 ± 0.05[a]	1.17 ± 0.05[b]	1.28 ± 0.05[a,b]
Live born (average)	1.38 ± 0.04[a]	1.19 ± 0.04[b]	1.25 ± 0.04[a,b]
Average crown-rump length, *cm*			
Female	26.9 ± 1.24	25.8 ± 1.28	23.6 ± 1.20
Males	27.3 ± 1.27	25.9 ± 1.31	23.8 ± 1.23
Intra-litter variability, *kg*[2]	0.20 ± 0.01	0.21 ± 0.02	0.21 ± 0.01

[1]Values are LSmeans ± SE; Labeled LSmeans (a, b $p < 0.05$) without a common letter differ within a row.

[2]Intra-litter variability of birth weight was calculated as square root of the pooled litter variance of male and female piglets within each litter (8).

Supplementary Table S4. Statistically non-significant ($p > 0.05$) body weight measurements, from weaning (28 d) to 56 d (prior to catheter implantation) measured in juvenile age class 80 d porcine offspring exposed to low (LP-HC; 1:10.4, $n = 25$), high (HP-LC; 1:1.3, $n = 27$), or adequate (AP; 1:5, $n = 25$) protein to carbo-hydrate ratio diets during gestation. Values are LSmeans ± SE.

		Maternal diet[1]		
Parameter	Age (d)	AP	LP-HC	HP-LC
Body weight, *kg*	28	7.62 ± 0.37	8.31 ± 0.33	7.85 ± 0.46
	35	8.50 ± 0.41	8.95 ± 0.36	8.30 ± 0.50
	42	11.0 ± 0.47	11.3 ± 0.42	10.6 ± 0.59
	49	14.7 ± 0.55	14.7 ± 0.49	13.8 ± 0.70
	56	19.0 ± 0.57	18.6 ± 0.51	17.1 ± 0.77

[1]Number of animals; AP = 27; LP-HC = 25; HP-LC = 26.

Supplementary Table S5. Birth weight, plasma and serum biochemical parameters of 1 and 80 d old porcine offspring exposed to low (LP-HC; 1:10.4), high (HP-LC; 1:1.3), or adequate (AP; 1:5) protein to carbohydrate ratio diets during gestation. Values are LSmeans ± SE.

		Maternal diet[1]			*P value*, ≤[2]
Parameters	Age class	AP	LP-HC	HP-LC	Age class
**Body weight, *kg***					
Birth	1 and 80 d	1.34 ± 0.04	1.21 ± 0.04	1.23 ± 0.04	0.93
**Metabolites**					
Glucose,	1 d	5.18 ± 0.26	4.94 ± 0.25	5.62 ± 0.25	0.06
mmol/L	80 d	5.57 ± 0.28	5.74 ± 0.29	5.68 ± 0.28	
NEFA,	1 d	242 ± 39	194 ± 38	285 ± 37	0.53
μmol/L	80 d	269 ± 40	292 ± 42	221 ± 41	
HDL cholesterol,	1 d	0.83 ± 0.09	0.91 ± 0.08	0.95 ± 0.11	0.08
mmol/L	80 d	1.23 ± 0.10	1.06 ± 0.10	0.88 ± 0.14	
LDL cholesterol,	1 d	1.13 ± 0.24	1.42 ± 0.20	0.94 ± 0.29	0.19
mmol/L	80 d	1.41 ± 0.23	1.43 ± 0.23	1.48 ± 0.30	
Total cholesterol,	1 d	2.14 ± 0.15	2.36 ± 0.15	2.47 ± 0.15	0.02
mmol/L	80 d	2.61 ± 0.16	2.59 ± 0.17	2.66 ± 0.16	
**Ratios**					
Glucose:Insulin[3]	1 d	0.25 ± 0.04	0.31 ± 0.04	0.18 ± 0.03	0.07
	80 d	0.21 ± 0.05	0.17 ± 0.05	0.18 ± 0.05	

[1]Number of animals; Age class 1 d (AP = 46–53; LP-HC = 47-49; HP-LC = 51–58), except for HDL and LDL cholesterol (AP = 21–23; LP-HC = 28; HP-LC = 15–18). Age class 80 d (AP = 24–27; LP-HC = 23-25; HP-LC = 22–26), except for HDL and LDL cholesterol (AP = 14; LP-HC = 14; HP-LC = 7).

[2]ANOVA F-Test.

[3]mmol/pmol.

Supplementary Table S6. Body weight, IVGTT analytes, glucose turnover and pools of 68 d old porcine offspring exposed to low (LP-HC; 1:10.4), high (HP-LC; 1:1.3), or adequate (AP; 1:5) protein to carbohydrate ratio diets during gestation. Values are LSmeans ± SE.

	Maternal diet[1]		
Parameter	AP	LP-HC	HP-LC
Number of piglets	27	25	27
Body weight, *kg*	22.8 ± 0.63	22.7 ± 0.56	20.7 ± 0.82
Fasting			
Glucagon, *pg/mL*	0.02 ± 0.002	0.02 ± 0.001	0.02 ± 0.002
Glucose, *mmol/L*	5.83 ± 0.11	5.84 ± 0.10	5.69 ± 0.15
Insulin, *μU/mL*	0.36 ± 0.06	0.35 ± 0.05	0.44 ± 0.08
AUC			
Glucose (2-30 min), *mmol/L · min*	59.7 ± 3.18	56.4 ± 2.80	66.6 ± 4.38
Insulin (0-30 min), *μU/mL · min*	434 ± 53	469 ± 47	550 ± 73
Glucose turnover, *g / kg BW / day*	15.0 ± 1.29	14.3 ± 1.13	14.4 ± 1.78
Glucose pool, *mg / kg BW*	426 ± 9	437 ± 8	431 ± 13

[1]Sex composition (AP; female = 17, male = 10, LP-HC; female = 12, male 13, HP-LC; female = 10, male = 17).

Supplementary Table S7. Body weight, urea turnover and pool of 76 d old porcine offspring exposed to low (LP-HC; 1:10.4), high (HP-LC; 1:1.3), or adequate (AP; 1:5) protein to carbohydrate ratio diets during gestation. Values are LSmeans ± SE.

	Maternal diet[1]		
Parameter	AP	LP-HC	HP-LC
Number of piglets	27	25	27
Body weight, kg	25.8 ± 0.73	25.9 ± 0.65	23.7 ± 0.93
Urea pool, *mg / kg BW*	114 ± 11	117 ± 10	97 ± 15
Urea turnover, *mg / kg BW / day*	428 ± 47	448 ± 42	285 ± 59

[1] Sex composition (AP; female = 17, male = 10, LP-HC; female = 12, male 13, HP-LC; female = 10, male = 17)

Supplementary Table S8. Plasma targeted metabolite ratios and sums of porcine offspring exposed to low (LP-HC; 1:10.4), high (HP-LC; 1:1.3), or adequate (AP; 1:5) protein to carbohydrate ratio diets during gestation.

		Maternal diet[1]			p value, ≤[2]	
Parameter[3]	Age class	AP	LP-HC	HP-LC	Age class	Diet*Age class
Number of piglets / age class		8	8	8		
**Ratios**						
C2 / C0	1	0.28 ± 0.02[ac]	0.21 ± 0.02[bc]	0.24 ± 0.02[c]	<0.001	0.03
	80	0.10 ± 0.01[d]	0.12 ± 0.02[d]	0.10 ± 0.02[d]		
Cit / Arg	1	0.81 ± 0.09	0.83 ± 0.09	0.88 ± 0.09	<0.001	0.95
	80	0.48 ± 0.09	0.52 ± 0.09	0.52 ± 0.09		
Cit / Orn	1	1.22 ± 0.13	1.32 ± 0.13	1.45 ± 0.13[c]	<0.001	0.24
	80	0.88 ± 0.13	1.03 ± 0.13	0.74 ± 0.13[d]		
Fischer's ratio	1	1.27 ± 0.18[d]	1.59 ± 0.18	1.26 ± 0.18[d]	<0.001	0.06
	80	2.92 ± 0.18[c]	2.35 ± 0.18	2.55 ± 0.18[c]		
Orn / Arg	1	0.65 ± 0.06	0.65 ± 0.06	0.62 ± 0.06	<0.001	0.19
	80	0.56 ± 0.06	0.55 ± 0.06	0.72 ± 0.06		
SDMA / Arg	1	0.02 ± 0.01	0.04 ± 0.01[c]	0.03 ± 0.01	<0.001	0.73
	80	0.01 ± 0.01	0.01 ± 0.01[d]	0.01 ± 0.01		
Spermidine / Putrescine	1	0.14 ± 0.07	0.22 ± 0.07	0.18 ± 0.07	0.038	0.99
	80	0.27 ± 0.07	0.36 ± 0.07	0.30 ± 0.07		
**Sums,** *(µM)*						
Aromatic AA	1	387 ± 32[c]	284 ± 32	382 ± 32[c]	<0.001	0.10
	80	177 ± 32[d]	208 ± 32	194 ± 32[d]		
Total SMOH	1	8.45 ± 0.78	9.48 ± 0.78	8.74 ± 0.78	0.01	0.39
	80	11.5 ± 0.78	10.4 ± 0.78	10.4 ± 0.78		

[1]Values are LSmeans ± SE; Labelled LSmeans ([a, b] $p < 0.05$) without a common letter differ within a row, ([c, d] $p < 0.05$) without a common letter differ within a column, based on Tukey post hoc analysis.

[2]ANOVA F-Test.

[3]Only significantly different parameters are shown. Abbreviations; ratios of acetylcarnitine to free carnitine (C2 / C0), citrulline to arginine (Cit / Arg), citrulline to ornithine (Cit / Orn), ornithine to arginine (Orn / Arg), symmetric dimethylated Arg to unmodified Arg pool (SDMA / Arg); sums of hydroxylated ceramide phosphocholines (sphingomyelins) (Total-SMOH).

Supplementary Figure S1. Intravenous glucose tolerance test conducted at 68 days of life in juvenile porcine offspring exposed to low (LP-HC; 1:10.4, *n* = 25), high (HP-LC; 1:1.3, *n* = 27), or adequate (AP; 1:5, *n* = 25) protein to carbohydrate ratio diets during gestation. Plasma concentrations of, **A** glucagon, and ratios of **B** glucose to insulin and **C** glucagon to insulin. Values are LSmeans ± SE. Diet, Time and the interaction of Diet x Time are ANOVA F-Test results.

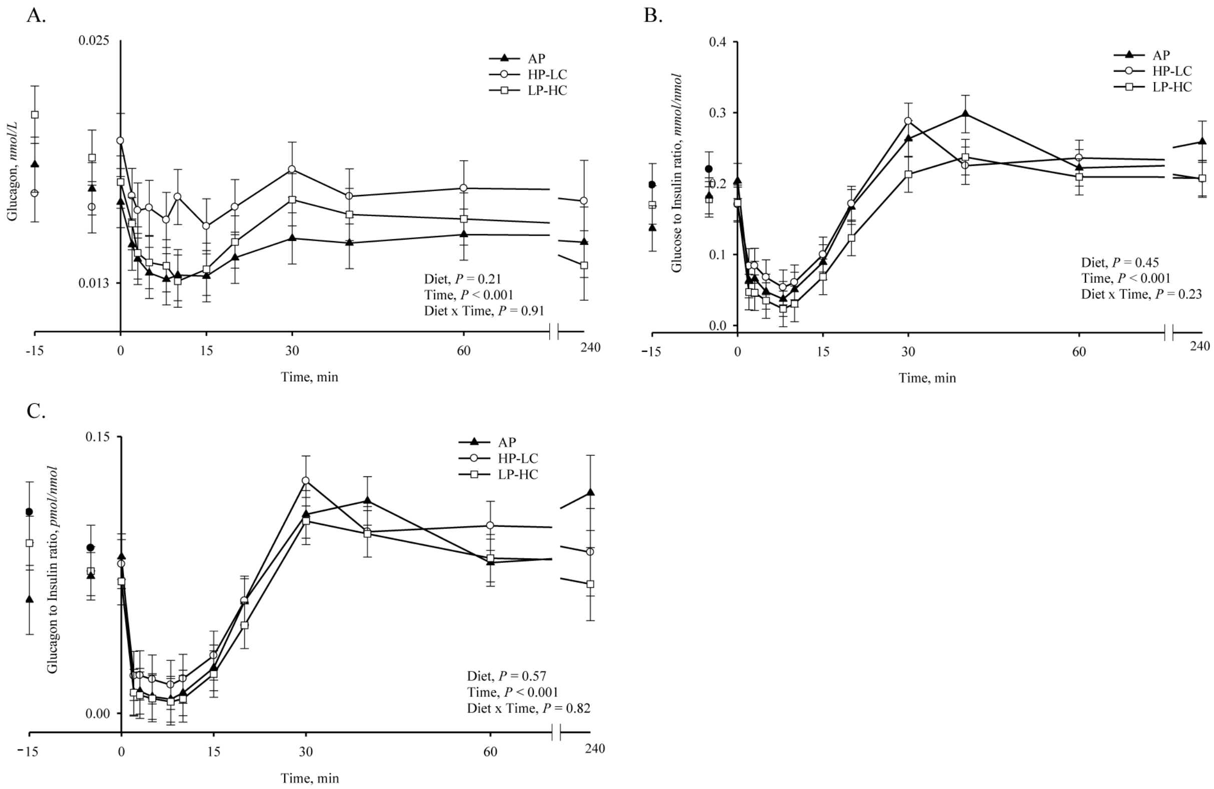

Supplementary Figure S2. Intravenous **i**nsulin challenge conducted in 71 day of juvenile porcine offspring exposed to low (LP-HC; 1:10.4), high (HP-LC; 1:1.3), or adequate (AP; 1:5) protein to carbohydrate ratio diets during gestation. Values are LSmeans ± SE. Diet and the interaction of Diet x Time are ANOVA F-Test results.

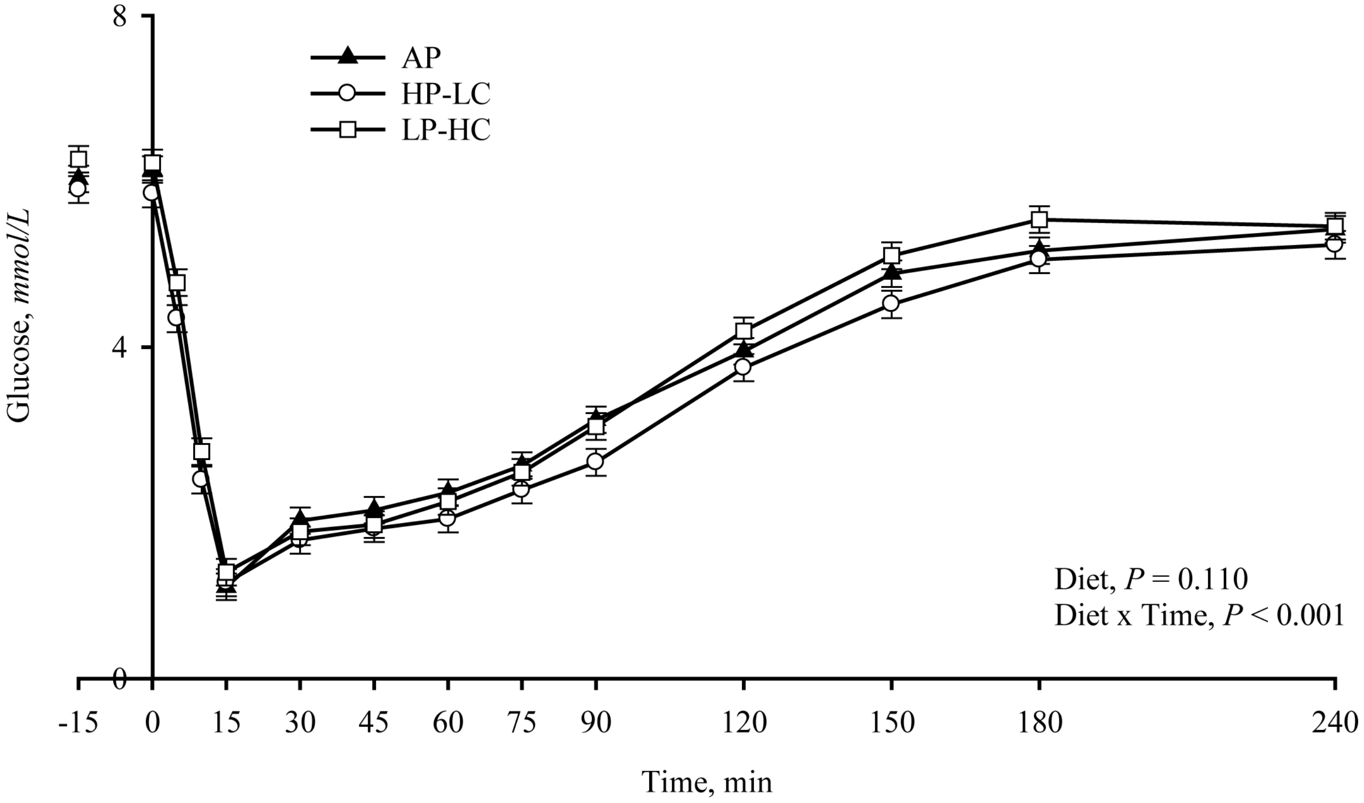

Supplement: Supplementary file 1 [file nutrients-13-03286-s001.zip › nutrients-1333259-supplementary.pdf]
